# A Variant in the Osteoprotegerin Gene Is Associated with Coronary Atherosclerosis in Patients with Rheumatoid Arthritis: Results from a Candidate Gene Study

**DOI:** 10.3390/ijms16023885

**Published:** 2015-02-11

**Authors:** Cecilia P. Chung, Joseph F. Solus, Annette Oeser, Chun Li, Paolo Raggi, Jeffrey R. Smith, C. Michael Stein

**Affiliations:** 1 Departments of Medicine and Biostatistics, Vanderbilt University, Nashville, TN 37232, USA; E-Mails: c.chung@vanderbilt.edu (C.P.C.); joseph.f.solus@vanderbilt.edu (J.F.S.); annette.oeser@vanderbilt.edu (A.O.); jeffrey.smith@vanderbilt.edu (J.R.S.); mike.stein@vanderbilt.edu (C.M.S.); 2 Biostatistics, Vanderbilt University, Nashville, TN 37232, USA; E-Mail: Chun.li3@case.edu; 3 Mazankowski Alberta Heart Institute, Department of Medicine, University of Alberta, Edmonton, AB T6G 2B7, Canada

**Keywords:** rheumatoid arthritis, genetic variation, SNP, atherosclerosis, rs2073618

## Abstract

Objective: Patients with rheumatoid arthritis (RA) have accelerated atherosclerosis, but there is limited information about the genetic contribution to atherosclerosis in this population. Therefore, we examined the association between selected genetic polymorphisms and coronary atherosclerosis in patients with RA. Methods: Genotypes for single-nucleotide polymorphisms (SNPs) in 152 candidate genes linked with autoimmune or cardiovascular risk were measured in 140 patients with RA. The association between the presence of coronary artery calcium (CAC) and SNP allele frequency was assessed by logistic regression with adjustment for age, sex, and race. To adjust for multiple comparisons, a false discovery rate (FDR) threshold was set at 20%. Results: Patients with RA were 54 ± 11 years old and predominantly Caucasian (89%) and female (69%). CAC was present in 70 patients (50%). A variant in rs2073618 that encodes an Asn3Lys missense substitution in the osteoprotegerin gene (*OPG*, *TNFRSF11B*) was significantly associated with the presence of CAC (OR = 4.09, *p* < 0.00026) and withstands FDR correction. Conclusion: Our results suggest that a polymorphism of the *TNFRSF11B* gene, which encodes osteoprotegerin, is associated with the presence of coronary atherosclerosis in patients with RA. Replication of this finding in independent validation cohorts will be of interest.

## 1. Introduction

Patients with rheumatoid arthritis (RA) have accelerated atherosclerosis, with a two-fold increased risk in cardiovascular events [1]. However, traditional cardiovascular risk factors and inflammatory markers do not fully explain this increased risk [2,3,4,5]. Therefore, efforts to identify additional risk factors and improve our ability to predict cardiovascular risk in this patient population are important.

Rheumatoid arthritis and atherosclerosis are chronic inflammatory conditions and both have significant genetic susceptibility. Because heritability accounts for up to 60% of the risk in RA and 30%–60% of the risk in cardiovascular disease [6] there has been increasing interest in attempting to identify genetic markers of atherosclerosis in RA.

Recent studies have described the role of a few single nucleotide polymorphisms (SNPs) in single genes, such as *MTHFR*, *TNFA*, and *ZC3HC1*, *NFKB* or other SNPs such as rs964184 on atherosclerosis in RA [7,8,9,10,11]. In this study, we undertook a more extensive candidate gene approach and selected polymorphisms located in 152 genes involved in inflammation, autoimmunity and cardiovascular disease (CVD). We examined the association of these selected genetic polymorphisms with coronary atherosclerosis in patients with RA.

## 2. Results

Patients with RA were 54 ± 11 years old on average and predominantly Caucasian (89%) and female (69%). Patients had a mean disease activity score based on the 28 joint count assessment (DAS28) of 3.6 ± 1.6 units and coronary calcium was detected in 70 patients (50%). Patients with coronary calcium were older, had longer disease duration, higher systolic blood pressure. There were more female patients in the group with coronary calcium than in the group without coronary calcium (Table 1).

**Table 1:** Clinical characteristics of patients with rheumatoid arthritis with and without coronary calcium.

Patient Characteristics	With Coronary Calcium (*n* = 70)	Without Coronary Calcium (*n* = 70)	*p*-Value
Age (years)	60 ± 9	48 ± 10	<0.001
Female sex (%)	59%	80%	0.01
Caucasian	90%	87%	0.40
Disease duration (years)	11 ± 11	6 ± 8	0.03
Systolic blood pressure	138 ± 21	129 ± 17	0.02
Diastolic blood pressure	76 ± 11	75 ± 10	0.24
Body mass index	29 ± 6	30 ± 7	0.64
Total cholesterol	184 ± 38	184 ± 41	0.67
LDL cholesterol	113 ± 27	109 ± 32	0.42

After adjustment for age, gender and race, SNPs in the genes tumor necrosis factor receptor superfamily member 11 b (*TNFRSF11B*), matrix metalloproteinase-3 (*MMP3*), interleukin-12 (*IL12*), matrix metalloproteinase-9 (*MMP9*), nucleotide-binding oligomerization domain-2 (*NOD2*), C-reactive protein (*CRP*), myeloperoxidase (*MPO*), resistin (*RETN*), interferon regulatory factor 5 (*IRF5*) and Fcχ receptor 2A (*FCGR2A*) were significantly associated with the presence of CAC (Table 2). *MMP9* contained more than one significant SNP; examination of pairwise LD (Linkage disequilibrium), defined as the correlations among neighboring alleles [12], between these is consistent with a single genetic effect per gene (pairwise *R*^2^ range 0.622–0.965 for all showing association). Strong linkage disequilibrium (LD) was observed between rs3918249 and rs17576 (chromosome 20, *R*^2^ = 0.965). All other pairs had an *R*^2^ < 0.8.

**Table 2:** Genetic association with coronary atherosclerosis in patients with rheumatoid arthritis (RA).

SNP	Gene	Major, Minor Allele	Minor Allele Frequency	Odds Ratio * (95% C.I.)	*p*-Value
rs2073618 **	*TNFRSF11B*	G,C	0.36	4.09 (1.93–8.70)	0.00026
rs522616	*MMP3*	T,C	0.29	4.43 (1.77–11.10)	0.001
rs2853694	*IL12B*	T,C	0.29	3.09 (1.53–6.24)	0.002
rs3918249	*MMP9*	T,C	0.49	0.36 (0.18–0.69)	0.002
rs17576	*MMP9*	A,G	0.45	0.34 (0.17–0.68)	0.002
rs3918253	*MMP9*	C,T	0.28	0.38 (0.20–0.72)	0.003
rs2274756	*MMP9*	G,A	0.16	0.24 (0.09–0.64)	0.004
rs751271	*NOD2*	T,G	0.49	2.57 (1.33–4.96)	0.005
rs650108	*MMP3*	G,A	0.41	2.82 (1.29–6.17)	0.009
rs1800947	*CRP*	C,G	0.04	5.93 (1.43–24.71)	0.014
rs9562414	*TNFSF11*	A,G	0.06	0.25 (0.08–0.79)	0.019
rs2107545	*MPO*	T,C	0.26	0.42 (0.20–0.89)	0.023
rs3745367	*RETN*	G,A	0.39	0.45 (0.22–0.91)	0.026
rs10954213	*IRF5*	G,A	0.47	0.50 (0.27–0.94)	0.030
rs633137	*TNFSF11*	T,A	0.08	0.34 (0.13–0.91)	0.031
rs2243828	*MPO*	A,G	0.23	0.43 (0.20–0.96)	0.040
rs1801274	*FCGR2A*	A,G	0.43	0.52 (0.28–1.00)	0.049

* Odds ratios for the comparison between minor and major allele; SNP = single-nucleotide polymorphism; C.I. = confidence interval; ** rs2073618 is the only SNP that remains significant after false discovery rate (FDR) correction.

Of all the variants initially associated with the presence of coronary atherosclerosis, rs2073618, which encodes an Asn3Lys missense change in the osteoprotegerin gene (*TNFRSF11B*), was most highly associated with the presence of coronary artery calcium (CAC) (OR = 4.09, *p* = 0.00026). This association remained after false discovery rate (FDR) correction.

**Figure 1:**
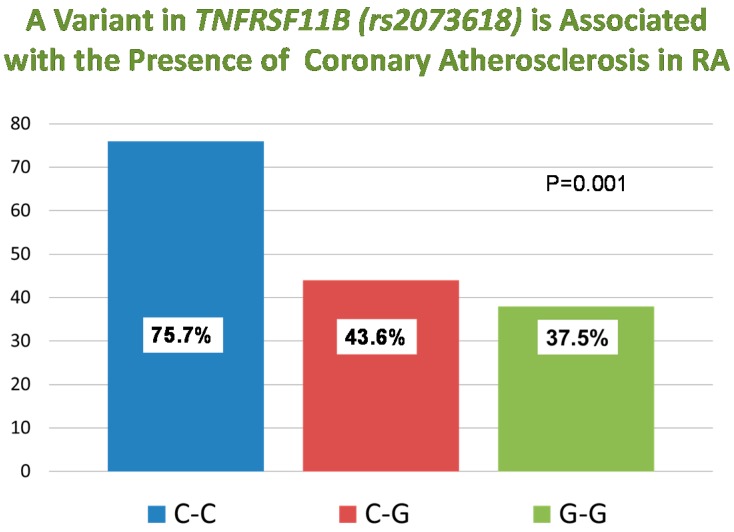
Prevalence of coronary calcium by genotype. The *p*-value was obtained using the Fisher’s exact test.

In the 139 patients in whom rs2073618 genotypes were available, 37 (26.6%) had the CC, 62 (44.6%) the CG, and 40 (28.8%) the GG genotypes. Figure 1 depicts the prevalence of coronary calcium by genotype. Among RA patients with CC genotype, 75.7% had coronary calcium; as compared with 43.6% in those with CG genotype and with 37.5% in those with GG genotype (*p* = 0.001). However, in a *post-hoc* analysis examining the association of rs2073618 genotypes and serum osteoprotegerin concentrations, differences were non-significant [13]. (Median (interquartile range, IQR) concentrations were 1548 (1042–2509), 1497 (1100–1698), and 1657 (1132–2105) pg/mL, respectively, *p* = 0.38).

## 3. Discussion

To the best of our knowledge, this is the first study examining the relationship between relevant published genetic variants in a range of genes of biological relevance to coronary atherosclerosis and inflammation in patients with RA. The major finding of this study is that a polymorphism of the *TNFRSF11B* gene, which encodes osteoprotegerin, is associated with the presence of coronary atherosclerosis in patients with RA.

Osteoprotegerin is a member of the TNF receptor family and acts as a soluble decoy receptor for receptor activator of nuclear factor KB ligand (RANKL) [14]. Initially, it was recognized as a mediator preventing osteoclast differentiation, activation and survival, and thus, a regulator of bone resorption [14]. More recently, in addition to its role in bone metabolism, increased osteoprotegerin concentrations have been associated with the presence and severity of atherosclerosis in the general population [15] and with inflammatory diseases, such as RA [16].

We previously showed that in patients with RA, the concentration of osteoprotegerin was higher than in control subjects, and increased osteoprotegerin concentrations were associated with both coronary artery calcification and sedimentation rate [13]. Furthermore, osteoprotegerin is associated with carotid atherosclerosis and endothelial activation in patients with RA [17]. Therefore, osteoprotegerin and the gene encoding it are potential mechanistic links between inflammation and atherosclerosis in RA [13].

In a recent study, Genre *et al.* examined the role of three functional polymorphisms (rs3134063, rs2073618 and rs3134069) located in the gene encoding osteoprotegerin. The study showed a protective effect of the osteoprotegerin (OPG) CGA haplotype on the risk of cardiovascular disease in patients who were anti-cyclic citrullinated peptide (anti-CCP) negative. Further examination of the role of individual SNPs suggested that the GG genotype in rs2073618 was associated with a significant reduction of cerebrovascular, but not cardiovascular events [18]. Concordant with our main findings in this study, two recent studies showed that the same polymorphism in the gene encoding osteoprotegerin, rs2073618, was associated with atherosclerosis in other patient populations. The same CC genotype was associated with a three-fold increased risk of stroke in patients with diabetes [19] and the frequency was two-fold higher in patients who underwent carotid endarterectomy compared to control subjects. This last association was even stronger when the results were adjusted for age, sex, hypertension, hypercholesterolemia, diabetes, coronary artery disease, peripheral artery disease and smoking [20].

However, despite consistent results showing that the CC genotype in rs2073618 confers an increased risk of atherosclerosis in different patient populations, the mechanisms underlying this association remain unclear. We and others have found that increased osteoprotegerin concentrations independently predict coronary atherosclerosis, endothelial activation, carotid atherosclerosis and cardiovascular disease [14,21,22]. Therefore, one possible explanation for our findings was that osteoprotegerin concentrations were affected by rs2073618 variants. However, that was not the case, as we did not find an association between osteoprotegerin concentrations and rs2073618 genotype. Other potential explanations could be hypothesized. For example, considering that this polymorphism causes a substitution from asparagine to lysine, it is possible that the variant alters function of the osteoprotegerin protein rather than its concentration. Both genome evolutionary rate profiling and functionally conserved sequence element alignments suggest that this substitution might be deleterious. Thus, this substitution may have an effect on either protein or transcriptional activity.

In addition to rs2073618, our data suggest that SNPs located in genes including *MMP3*, *IL12*, *MMP9*, *NOD2*, *CRP*, *MPO*, *RETN*, *IRF5*, and *FCGR2A* could be of interest. Although the results did not remain significant after adjustment for multiple comparisons, the magnitude of the effect appears large.

Other genetic variants, such as the rheumatoid arthritis shared epitope, have been proposed as markers of endothelial dysfunction and coronary atherosclerosis in RA [23,24,25]. Our study did not find an association between the SNPs tagging *HLA-DRB-1* and *CAC*. In concordance with our other findings, there are reports of no association between genetic variants in genes regulating IL-1, interferon-gamma, toll like receptor-4, and IL-6 and atherosclerosis in RA [26,27,28,29]. Most of these studies were limited to examining single genetic variants.

Recent studies in other populations also examined the association between multiple genetic variants and CAC. A meta-analysis of genome-wide association studies for CAC in community cohort studies identified SNPs near *CDKN2A* and *CDKN2B*, *PHACTR1*, *MRAS*, *COL4A1*/*COL4A2*, and *SORT1* as significantly associated with CAC [30]. Another study, using a candidate gene approach to evaluate patients with chronic kidney disease, identified rs13260 (*COL4A1*) and rs7964239 (*BCAT1*) as SNPs associated with the presence of coronary calcium scores >0 [31], but because the selection of our candidate SNPs was done before these studies were published, we did not examine them.

Our results need to be interpreted in the light of some limitations. First, the sample size is small for genetic association studies and our results should be seen as hypothesis generating. Although rs2073618 reached the predefined 20% FDR threshold adjustment for multiple comparisons, it is possible that some other relevant SNPs did not reach statistical significance due to limitations in power. Second, this study examined the role of individual SNPs as markers for atherosclerosis; but the development of risk scores with multiple validated SNPs may have even better potential as tool to improve cardiovascular risk estimation. Finally, given the potential for false positive associations [32] and noted differences with the study by Genre *et al.* [18] replication of our findings in other cohorts will be necessary.

## 4. Methods

One hundred and forty patients with RA, who have participated in studies to evaluate the prevalence and risk factors of coronary atherosclerosis in RA, were included. Details of the protocol have been described previously [3]. In summary, patients fulfilled the 1987 American College of Rheumatology classification criteria for RA [33] and were 18 years or older. Clinical information was obtained by medical record review, structured interview, and physical examination. Blood was collected and erythrocyte sedimentation rate (ESR) measured, and the disease activity score based on the 28 joint count assessment (DAS28) [34] was calculated.

As described previously, [3] the presence of coronary artery calcium (CAC), a non-invasive measurement of coronary atherosclerosis, was detected by electron beam computed tomography (EBCT) scan with an Imatron C-150 scanner (GE/Imatron, South San Francisco, CA, USA). All scans were read by a single investigator (PR), who was unaware of the subjects’ clinical status.

After review of the literature, 653 SNPs in 152 genes involved in inflammation, CVD and autoimmune diseases (see Table S1), were selected. Tagging SNP markers were selected based upon HapMap CEU subject data (*R*^2^ ≥ 0.8, maf > 0.05) for genes encompassing 3–20 kb upstream to 1–10 kb downstream of each respective coding region or were represented by a single SNP selected from published disease association studies. Deoxyribonucleic acid (DNA) was extracted from blood samples using Gentra Puregene DNA Purification reagents and standard protocols on an Autopure LS instrument (Qiagen, Valencia, CA, USA). Quantification was done with a Nanodrop 2000 instrument (Thermo Scientific, Waltham, MA, USA). Samples were diluted to 50 ng/µL and genotyping was done by GoldenGate assay on a Beadstation 500GX instrument (Illumina Corp, San Diego, CA, USA); 7.5% of the SNPs failed in genotyping or were monomorphic in our RA population and were not included in analysis. No SNPs were excluded for deviation from Hardy–Weinberg Equilibrium. All samples were called in >90% of the remaining SNPs and were included in subsequent analysis.

The association between rs2073618 genotypes and osteoprotegerin concentrations was examined in a *post-hoc* analysis using the Kruskal–Wallis test. As previously reported [13], serum osteoprotegerin was measured using enzyme-linked immunosorbent assay method using commercial kits (R & D Systems, Minneapolis, MN, USA).

Baseline characteristics in patients with RA are presented as mean (±SD), median (interquartile range), or frequency (%). Baseline characteristics between patients with and without coronary calcium were compared with the use of Wilcoxon rank sum test or with Fisher’s exact test, as appropriate. The association between the presence of CAC and individual SNPs was assessed with logistic regression with an additive effect for each allele. Results were adjusted for age, sex and race, covariates that were chosen a priori. A two-sided *p*-value of <0.05 was considered significant. To adjust for multiple comparisons, a pre-specified false discovery rate (FDR) threshold was set at 20%.

We conducted the analyses using STATA 12.0 (Stata Corp., College Station, TX, USA). SNAP (SNP annotation and proxy search; www.broadinstitute.org) was used to estimate pair-wise linkage disequilibrium (LD) between individual SNPs within the same gene.

All study participants provided written informed consent and the study was approved by the Institutional Review Board of Vanderbilt University on 10 August 2014 (IRB# 000567).

## Supplementary Materials

Supplementary materials can be found at http://www.mdpi.com/1422-0067/16/02/3885/s1.
